# Reply to: Efficacy and safety of high-flow nasal cannula oxygen therapy in moderate acute hypercapnic respiratory failure

**DOI:** 10.5935/0103-507X.20200035

**Published:** 2020

**Authors:** Maria Eugenia Yuste, Olga Moreno, Susana Narbona, Fernando Acosta, Luis Peñas, Manuel Colmenero

**Affiliations:** 1 Unidade de Terapia Intensiva, Hospital Universitario San Cecilio - Granada, Espanha.; 2 Instituto de Investigación Biosanitaria - Granada, Espanha.

**To the Editor**

We thank Dr. Esquinas and Reazaul for their interest in our study and their thoughtful commentaries about some aspects of the methodology and the generalizability of the results.

The protocol of the use of high-flow nasal cannula (HFNC) was extracted from published studies on hypoxemic failure.^(1)^High-flow nasal cannula was used continuously, without interruptions, including nighttime until gas exchange and respiratory distress were reversed. Parameters of HFNC were adjusted depending on oxygen saturation (fraction of inspired oxygen) and respiratory rate (flow), as other more specific surrogates of breathing effort (esophageal pressure-time product) were not measured.^(2)^

Regarding the types of patients with hypercapnia, they are described as a mixed population of chronic obstructive pulmonary disease (COPD), congestive heart failure and sleep-related or obesity hypoventilation. A large proportion (20 out of 30) became hypercapnic after being ventilated and extubated for reasons not related to their comorbid condition. Therefore they are not exclusively hypercapnic failure due to acute on chronic decompensation of COPD patients. This is why we do not restrict our conclusions to a specific patient population or disease, but to deranged physiology leading to hypercapnia.

You can get the data about time to control hypercapnia and clinical improvement from our results (Table 1 - Clinical and gas exchange parameters, p. 159). What we can not draw any conclusion is about the successful management of patients with noninvasive mechanical ventilation after HFNC failure, because there was only one patient in this category.^(3)^

As we have stated in the manuscript, the results of our study due to its observational nature and small sample size, have to be considered preliminary and need to be tested in rigorous clinical trials.^(4,5)^Nonetheless, we think they represent one of the first series of successful use of this therapy in patients with hypercapnia. We only advocate the use of HFNC in patients with moderate hypercapnia that do not tolerate or have contraindications for non-invasive ventilation. So far, this ultimate mode has to remain the standard and first-line ventilatory treatment for hypercapnic respiratory failure.^(6)^

## References

[1] Artacho Ruiz R, Artacho Jurado B, Caballero Güeto F, Cano Yuste A, Durbán García I, García Delgado F (2019). Predictors of success of high-flow nasal cannula in the treatment of acute hypoxemic respiratory failure. Med Intensiva.

[2] Rittayamai N, Phuangchoei P, Tscheikuna J, Praphruetkit N, Brochard L (2019). Effects of high-flow nasal cannula and non-invasive ventilation on inspiratory effort in hypercapnic patients with chronic obstructive pulmonary disease: a preliminary study. Ann Intensive Care.

[3] Yuste ME, Moreno O, Narbona S, Acosta F, Peñas L, Colmenero M (2019). Efficacy and safety of high-flow nasal cannula oxygen therapy in moderate acute hypercapnic respiratory failure. Rev Bras Ter Intensiva.

[4] Ricard JD, Dib F, Esposito-Farese M, Messika J, Girault C, REVA network (2018). Comparison of high flow nasal cannula oxygen and conventional oxygen therapy on ventilatory support duration during acute-on-chronic respiratory failure: study protocol of a multicentre, randomised, controlled trial. The 'HIGH-FLOW ACRF' study. BMJ Open.

[5] Thille AW, Muller G, Gacouin A, Coudroy R, Decavèle M, Sonneville R, Beloncle F, Girault C, Dangers L, Lautrette A, Cabasson S, Rouzé A, Vivier E, Le Meur A, Ricard JD, Razazi K, Barberet G, Lebert C, Ehrmann S, Sabatier C, Bourenne J, Pradel G, Bailly P, Terzi N, Dellamonica J, Lacave G, Danin PÉ, Nanadoumgar H, Gibelin A, Zanre L, Deye N, Demoule A, Maamar A, Nay MA, Robert R, Ragot S, Frat JP, HIGH-WEAN Study Group and REVA Research Network (2019). HIGH-WEAN Study Group and REVA Research Network: Effect of postextubation high-flow nasal oxygen with noninvasive ventilation vs high-flow nasal oxygen alone on reintubation among patients at high risk of extubation failure: a randomized clinical trial. JAMA.

[6] Rochwerg B, Brochard L, Elliott MW, Elliott MW, Hess D, Hill NS, Nava S, Navalesi P, Antonelli M, Brozek J, Conti G, Ferrer M, Guntupalli K, Jaber S, Keenan S, Mancebo J, Mehta S, Raoof S, Members of The Steering Committee, Members of The Task Force (2017). Official ERS/ATS clinical practice guidelines: noninvasive ventilation for acute respiratory failure. Eur Respir J..

